# Releasing the restraints of Vγ9Vδ2 T-cells in cancer immunotherapy

**DOI:** 10.3389/fimmu.2022.1065495

**Published:** 2023-01-13

**Authors:** Laura A. Ridgley, Jonathan Caron, Angus Dalgleish, Mark Bodman-Smith

**Affiliations:** Institute for Infection and Immunity, St. George’s University of London, London, United Kingdom

**Keywords:** Vγ9Vδ2 T-cell, BCG, ZA, immune checkpoint inhibitor, NKG2A

## Abstract

**Objectives:**

Vγ9Vδ2 T-cells are a subset of T-cells with a crucial role in immunosurveillance which can be activated and expanded by multiple means to stimulate effector responses. Little is known about the expression of checkpoint molecules on this cell population and whether the ligation of these molecules can regulate their activity. The aim of this study was to assess the expression of both activatory and inhibitory receptors on Vγ9Vδ2 T-cells to assess potential avenues of regulation to target with immunotherapy.

**Methods:**

Expression of various activatory and inhibitory receptors was assessed on Vγ9Vδ2 T-cells by flow cytometry following activation and expansion using zoledronic acid (ZA) and Bacillus Calmette-Guérin (BCG). Expression of these markers and production of effector molecules was also examined following co-culture with various tumour cell targets. The effect of immune checkpoint blockade on Vγ9Vδ2 T-cells was also explored.

**Results:**

Vγ9Vδ2 T-cells expressed high levels of activatory markers both at baseline and following stimulation. Vγ9Vδ2 T-cells expressed variable levels of inhibitory checkpoint receptors with many being upregulated following stimulation. Expression of these markers is further modulated upon co-culture with tumour cells with changes reflecting activation and effector functions. Despite their high expression of inhibitory receptors when cultured with tumour cells expressing cognate ligands there was no effect on Vδ2+ T-cell cytotoxic capacity or cytokine production with immune checkpoint blockade.

**Conclusions:**

Our work suggests the expression of checkpoint receptors present on Vγ9Vδ2 T-cells which may provide a mechanism with the potential to be utilised by tumour cells to subvert Vγ9Vδ2 T-cell cytotoxicity. This work suggests important candidates for blockade by ICI therapy in order to increase the successful use of Vγ9Vδ2 T-cells in immunotherapy.

## Introduction

The γδ T-cell is a unique cell population making up 1-5% of peripheral blood T-cells (1, 2). In contrast to the αβ T-cell the γδ T-cell comprises of a TCR made of a variable (V) γ chain and Vδ chain. There are numerous subsets of γδ T-cell with the main subsets being the Vδ1, Vδ2 and Vδ3 T-cells. The Vδ1 and Vδ3 subsets are most abundant in the intestinal mucosa whereas the most predominant subtype in the blood is the Vγ9Vδ2 T-cell (Vδ2) which is important in immunosurveillance against infection, for example *Mycobacterium tuberculosis*, *Listeria monocytogenes* and *Salmonella enterica* (3–5). This cell population has also been implicated in anti-tumour responses due to their ability to recognise phosphoantigens from dysregulated mevalonate pathways. Full activation occurs *via* the recruitment of butyrophilin 3A1 (BTN3A1), which together with BTN2A1 engages the T-cell receptor (TCR) (6–10). In addition to recognising phosphoantigens, Vγ9Vδ2 T-cells can also recognise upregulated cell stress ligands through expression of various NK associated activatory receptors (11). Confirming their role in immunosurveillance, the presence of γδ T-cells in tumours has been shown to correlate with clinical outcome in different cancer types (12–14). Genetic signatures reveal the γδ T-cell as the most significantly associated with favourable prognosis (15). Furthermore, high levels of circulating γδ T-cells have been associated with reduced cancer risk and improved survival (15–17).

Due to their inherent killing capacity these cells are promising tools for use in cancer immunotherapy. In the initial exploration into the use of these cells for immunotherapy studies utilized the expansion of γδ T-cells with various phosphoantigen derivates and nitrogen containing bisphosphonates, including zoledronic acid (ZA). Multiple trials have been conducted utilising *in vivo* expansion or *in vitro* expansion followed by adoptive transfer, showing varying degrees of success (18–25). Protocols for expansion of Vδ1 cells include IL-15, IL-7 and phytoheamagglutinin (PHA) or antigen presenting cells (APCs) expressing CD86, 41BBL, CD40L and cytomegalovirus (CMV)-antigen-pp65 (26–28). In contrast protocols most commonly used for expansion of Vδ2 cells include ZA, bromohydrin pyrophosphate (BrHPP) and (E)-4-hydroxy-3-methyl-but-2-enyl pyrophosphate (HMBPP) (22, 29). Potential explanations as to their varying efficacy include anergy, reduced migratory capacity and subsequent infiltration into tumours or high degree of polyclonality resulting in a diverse product.

Other candidates for expansion of Vδ2 T-cells include viruses and bacteria such as Bacillus Calmette-Guérin (BCG), the strain of mycobacterium used in the prevention of tuberculosis and in the treatment of bladder cancer (13, 30–32). BCG injection into melanoma lesions has resulted in regression of lesions and infiltration of IFN-γ-producing Vδ2 T-cells (33). Further support for the use of BCG in Vδ2 T-cell expansion comes as this method has been shown *in vitro* to result in altered cytolytic profiles compared to expansion using ZA (34).

In recent years several additional strategies have been utilized in order to improve γδ T-cell therapy (35, 36). These include antibody therapy, for example, agonistic monoclonal antibodies against BTN3A1, such as ICT01 in phase I/II clinical trial (NCT04243499), bispecific antibodies targeting TCR and tumour antigens, such as Vγ9-TCR engagers against HER2, CD1d and PSMA in clinical trials (NCT04887259 and NCT05369000) and bispecific Vγ9 nanobody-based constructs targeting EGFR in pre-clinical development (37–41). Other strategies focusing on cell therapy include chimeric antigen receptor (CAR)-transduced Vδ2 cells or αβ-T-cells transduced with Vγ9Vδ2 TCR, also called T-cells engineered with defined γδ TCR (TEG) (42–47).

Tumours have many mechanisms of evading the immune system, a key mechanism being the expression of ligands to checkpoint receptors expressed on effector immune cells. Tumours can display, amongst others, increased programmed death ligand 1 (PDL1) and HLA class I histocompatibility antigen alpha chain E (HLA-E), which bind programmed cell death protein 1 (PD1) and CD94/NK group 2 member A (NKG2A) to dampen T-cell and natural killer (NK) cell responses respectively (48). Further immune checkpoints include lymphocyte activation gene 3 (LAG3), T-cell immunoglobulin domain and mucin domain 3 (TIM3), T-cell immunoreceptor with Ig and ITIM domains (TIGIT), B and T lymphocyte attenuator (BTLA) which bind to their ligand’s major histocompatibility complex (MHC) class II, Galectin 9 (Gal9), poliovirus receptor (PVR) and herpesvirus entry mediator (HVEM) respectively. Understanding of immune checkpoints in relation to Vδ2 T-cells is currently limited. In a clinical setting, treatment with ipilimumab, an antibody targeting CTLA-4, increases Vδ2 T-cell number indicating a role for checkpoints in Vδ2 T-cell function (16). Confirming the importance of checkpoint receptors on Vδ2 T-cells a recent study documents increased proportions of Vδ2 T-cells expressing LAG3 in melanoma patients, a finding which was associated with earlier relapse and shorter overall survival (17). Upregulation of PD1 expression has been documented on Vδ2 T-cells following antigenic stimulation followed by a gradual decline (49). Furthermore, expression of PD1 is high on γδ T-cells from a variety of tumour types (50–52). Blockade of PD1 was able to enhance cytotoxicity and IFN-γ production although other studies have shown no effect of blockade on γδ T-cell function (49, 53, 54) Moreover, TIM3 co-expression with PD1 has been shown to result in lower IFN-γ and TNF production (55). Interaction with Gal9 lowered Vδ2 T-cell cytotoxicity by limiting perforin and Granzyme B (56). Furthermore, anti-TIM3 was able to enhance anti-tumour activity *via* increased cytokine production, this effect was independent of PD1 blockade suggesting a complex interplay of receptors will be important in Vδ2 T-cells activity (55).

The aim of this study was to investigate further the expression of activatory and inhibitory immune receptors on Vδ2 T-cells to determine the hierarchy of importance of these molecules in Vδ2 T-cell function and the importance of stimulation conditions on activation of Vδ2 T-cells. This will provide crucial information on the use of checkpoint inhibitor therapy alongside Vδ2 T-cell therapy.

## Materials and methods

### Subjects

Donor blood was obtained from healthy volunteers of leukocyte reduction system (LRS) cones from the National Health Service Blood and Transplant Unit (NHSBT) at St. George’s Hospital London under ethical approval SGREC16.0009.

### PBMC and Vγ9Vδ2 T-cell isolation

Peripheral blood mononuclear cells (PBMC) were isolated from LRS cones using density centrifugation over Histopaque-1077 (Sigma) as per manufacturer’s instructions. Erythrocytes were lysed using RBC lysis buffer (BioLegend) and platelets removed by centrifugation at 200g. PBMCs were stored at -80°C in freezing medium (45% RPMI-1640, 45% FBS, 10% DMSO). Following expansion γδ T-cells were resuspended in MACS buffer (PBS containing 0.5% BSA and 2mM EDTA) and isolated by negative enrichment using magnetic γδ T-cell negative selection kit (Miltenyi Biotech), according to manufacturer’s instructions. Isolated cells had median purities of >90%.

### Expansion and culture of Vγ9Vδ2 T-cells

For expansion of Vγ9Vδ2 T-cells thawed PBMCs were cultured at 5x10^6^ cells/ml in RPMI-1640 + 10% Foetal Bovine Serum (FBS; Sigma) with final concentrations of 10µM ZA (Sigma) or 2x10^4^ CFU BCG (Pasteur strain, gifts of Dr Rajko Reljic St. George’s University of London) both with 15ng/ml IL-2 (R&D Systems) in 200µl total volume in 96 well round bottomed plates. BCG was cultured as previously described (34) and in some cases, where indicated, BCG was heat killed by heating to 80°C for 30 minutes. Cells were cultured at 37°C with 5% CO_2_ for 14 days with media containing 15ng/ml IL-2 refreshed every 2-3 days.

### Tumour cell culture

Burkitt’s Lymphoma B-cell lines Daudi and Raji, and acute monocytic leukemia cell line THP-1, all from the European Collections of Authenticated Cell Cultures (ECACC), were cultured in RPMI-1640 + 10% FBS at 1x10^6^ per ml of 75cm^2^ tissue culture flask (Thermo Fisher Scientific). Cells were passaged every 2-4 days to maintain recommended cell densities and cells were used between passage 5-15. In some cases, prior to culture with γδ T-cells tumour cells were cultured with final concentration of 50µM ZA for 24 hours.

### Cytotoxicity assay

For cytotoxicity assays tumour cells were labelled with a final concentration of 0.5μM Cell Trace Far Red (Thermo Fisher Scientific) prior to culture. ZA or BCG stimulated γδ T-cells were co-cultured with Daudi, Raji or THP-1 cells, pre-treated with or without 50μM ZA, at an optimised effector:target cell ratio of 1:1. Cells were cultured in a total volume of 200µl in 96 well plates at 0.5x10^6^ cells/ml in RPMI + 10% FBS for 18 hours before being stained with zombie aqua, as described in Multiparameter Flow Cytometry. Specific killing was calculated by subtracting the dead cell frequency of targets cultured alone from the dead cell frequency of those in co-culture.

### CD107b mobilisation assay

For CD107b mobilisation assays γδ T-cells were cultured with tumour cells as previously described for cytotoxicity assay. Cells were cultured for 1 hour alone or with 25ng/ml Phorbol 12-myristate 13-acetate (PMA) and 1μg/ml ionomycin used as a positive control, before the addition of 5µg/ml Brefeldin A (BFA) and 2µM monensin (both Sigma Aldrich) and CD107b-FITC (H4B4; BioLegend) for 3 hours. Cells were harvested and stained for flow cytometry.

### TNF release assay

For TNF release assays γδ T-cells were cultured with tumour cells as previously described for cytotoxicity assay. Cells were cultured for 15 minutes before the addition of 10µM TAPI-0 (TNF-α protease inhibitor 0; Biotechne) and TNF-PECy7 (MAb11; Biolegend). Cells were cultured for 4 hours before being harvested and stained for flow cytometry.

### Immune checkpoint blockade

For the blockade of immune checkpoints in cytotoxicity and CD107b mobilisation assays the following antibodies were used: anti-IgG1, anti-IgG2, anti-PD1, anti-TIGIT, anti-LAG3, anti-TIM3 and anti-BTLA (all BioLegend). Anti-NKG2A antibodies were developed using a plant manufacturing system (57). All antibodies were used at final concentrations of 5µg/ml.

### Multiparameter flow cytometry

Cells were stained with Zombie Aqua viability dye (BioLegend) in PBS, according to manufacturer’s instructions, prior to antibody staining. Staining was performed in FACS buffer (PBS containing 2.5% BSA, 0.1% sodium azide and 2mM EDTA) for 30 minutes at 4°C. Cells were stained with the following antibodies as indicated CD3-BUV395 (UCHT1), CD56-BUV737 (NCAM16.2; both BD Biosciences) Vδ2-PE, Vδ2-PerCP-Vio700 (both REA771), PD1-VioBrightFITC (PD1.3.1.3), NKG2A-VioFITC (REA110), NKG2C-PE (REA205; all Miltenyi Biotech), CD3-AF700 (OKT3), BTLA-PE (MIH26), TIGIT-BV421 (A15153G), LAG3-BV711 (11C3C65), TIM3-BV605 (F38-2E2), NKG2D-APC (1D11), DNAM1-BV711 (11A8), KLRG1-BV421 (14C2A07), NKp44-PE (P44-8), NKp30-BV711 (P30-15), NKp46-BV421 (9E2; all BioLegend) and VISTA-APC (B7H5DS8; eBioscience). Cells were fixed with cell fix (BD Biosciences) prior to acquisition.

For intracellular staining cells were stained with Zombie Aqua prior to staining with surface antibodies CD3-BUV395 and Vδ2-PerCP-Vio700. Cells were fixed and permeabilised with fixation and permeabilization buffer (BioLegend), according to manufacturer’s instructions, and stained with IFN-γ-BV421 (4S.B3), TNF-BV711 (MAb11), Granzyme B-APC (QA16A02), Perforin-PE-Cy7 (B-D48), Granulysin-PE (DH2; all BioLegend).

Data was collected on a Fortessa X20 (BD Biosciences) and analysed using FlowJo (Treestar), using fluorescence-minus-one (FMO) gating. Debris was excluded by SSC-A versus SSC-W and live cells were gated based on exclusion of zombie aqua viability dye. Vγ9Vδ2 cells, T-cells and NK cells were gated as Vδ2+, CD3+ and CD56+ respectively and activatory and inhibitory receptor expression further examined, gating strategy as depicted in **Supplementary Figure 1**.

### Gene expression of Vγ9Vδ2 T-cells

RNA was extracted from Vγ9Vδ2 T-cells using RNeasy Micro Kit (Qiagen) as per manufacturer’s instructions. The purity of the isolated mRNA was assessed using a NanoDrop™ 2000 spectrophotometer (Thermo Fisher Scientific) and the quality and integrity using an Agilent 2100 Bioanalyser (Agilent Technologies). mRNA library was then prepared with the NEBNext Ultra II kit (New England Biolabs) and sequenced with a NextSeq 550 system (Illumina). Raw data was processed and analysed using Partek Flow (Partek).

### Statistics

Statistical analysis was performed using GraphPad prism 9 (GraphPad Software Inc). Non-parametric analysis of variance with Sidak *post hoc* pairwise analyses, non-parametric mixed effects analysis with Tukey’s *post hoc* pairwise analyses or non-parametric analysis of variance (Friedman’s) with Dunn’s *post hoc* for multiple pairwise comparisons carried out where indicated. P-values of <0.05 were considered statistically significant.

## Results

### Vγ9Vδ2 T-cells express NK associated activatory receptors and inhibitory checkpoint receptors

First, we aimed to assess the expression of NK associated activatory markers and inhibitory checkpoint receptors in Vδ2+ T-cells in freshly isolated PBMCs, gating strategy in **Supplementary Figure 1**.

**Figure 1:**
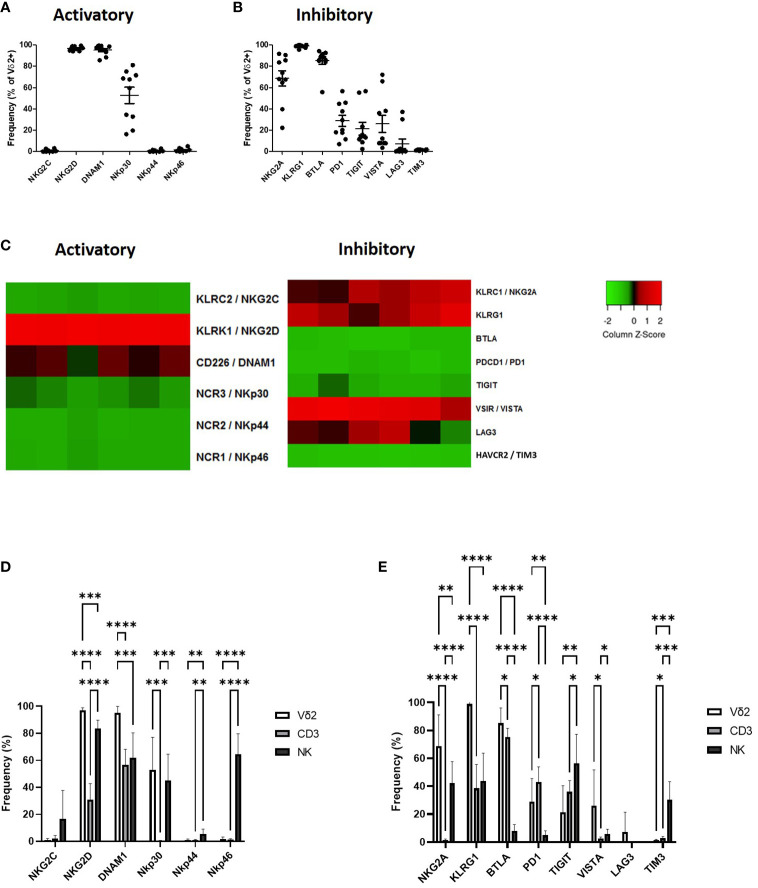
Expression of NK associated activatory receptors **(A)** and inhibitory checkpoint receptors **(B)** was determined in Vγ9Vδ2+ cells in PBMCs from healthy donors using flow cytometry. Heat maps showing the expression of NK associated activatory and inhibitory receptors in Vγ9Vδ2+ cells from 6 donors using RNAseq **(C)**. Expression of NK associated activatory receptors **(D)** and inhibitory checkpoint receptors **(E)** in Vγ9Vδ2+ cells was compared to CD3+ T-cells and CD56+ NK cells. N=10. *p<0.05, **p<0.005, ***p<0.0005, ****p<0.0001, non-parametric analysis of variance with Tukey’s *post hoc* for multiple pairwise comparisons.

Vδ2+ T-cells express several activatory receptors including NKG2D, DNAM1 and NKp30 (**Figure 1A**; **Supplementary Figure 2A**). Vδ2+ T-cells express a high level of inhibitory checkpoint receptors NKG2A, KLRG1 and BTLA, intermediate levels of PD1, TIGIT and VISTA and very little expression of LAG3 and TIM3 (**Figure 1B**; **Supplementary Figure 2B**).

The expression profile of Vδ2+ T-cells as seen by flow cytometry was largely confirmed by RNAseq. Expression of activatory marker NKG2D was particularly high with higher expression of DNAM1 and NKp30 also seen (**Figure 1C**). In terms of inhibitory receptors KLRG1, NKG2A and VISTA were confirmed at having higher expression at the gene level (**Figure 1C**).

The expression of NK-associated activatory and inhibitory checkpoint receptors on Vδ2+ T-cells in circulation confirms previous descriptions of these cells as a bridge between the innate and adaptive immune systems, therefore we assessed the expression of these molecules in comparison to CD3+ T-cells and CD56+ NK cells. Vδ2+ T-cells express similar levels of activatory receptors NKp30 compared to CD56+ NK cells from PBMC, however unlike NK cells Vδ2+ T-cells lack expression of NKp44 and NKp46 and have significantly higher expression of NKG2D and DNAM1 (**Figure 1D**). Compared to NK cells Vδ2+ T-cells express significantly higher levels of NKG2A, KLRG1, BTLA and PD1 and significantly lower levels of TIGIT and TIM3 (**Figure 1E**). In comparison with CD3+ T-cells Vδ2+ T-cells express significantly higher NKG2D, DNAM1 and NKp30 (**Figure 1D**). In addition, Vδ2+ T-cells express significantly higher NKG2A, KLRG1, BTLA and VISTA but significantly lower PD1 and TIM3 compared to CD3+ T-cells (**Figure 1E**).

The receptor expression profile of Vδ2+ T-cells is unique, highlighting the role of these cells as a bridge between the innate and adaptive immune response. This receptor profile provides both a mechanism of recognition and a potential method of regulation of Vγ9Vδ2 T-cells therefore, it will be important to know how the expression of these inhibitory markers is altered following activation as these may provide a mechanism of damping Vδ2+ T-cell response.

### Expression of inhibitory immune checkpoint receptors increases upon Vδ2+ T-cell activation

After showing the expression of a wide range of activatory and inhibitory immune receptors on Vδ2+ T-cells in circulation the next aim was to assess whether Vδ2+ T-cell stimulation resulted in alteration of these molecules. We hypothesised that an upregulation in the expression of inhibitory molecules following stimulation will provide a mechanism by which Vδ2+ T-cells can be restrained, with implications on their efficacy in immunotherapy.

The expression of activatory and inhibitory receptors was explored following 24 hours activation of PBMC. Isolated PBMCs were stimulated with IL-2 with and without previously optimised concentrations of ZA or BCG for 24 hours (34). Expression of activatory and inhibitory markers was assessed by flow cytometry.

**Figure 2:**
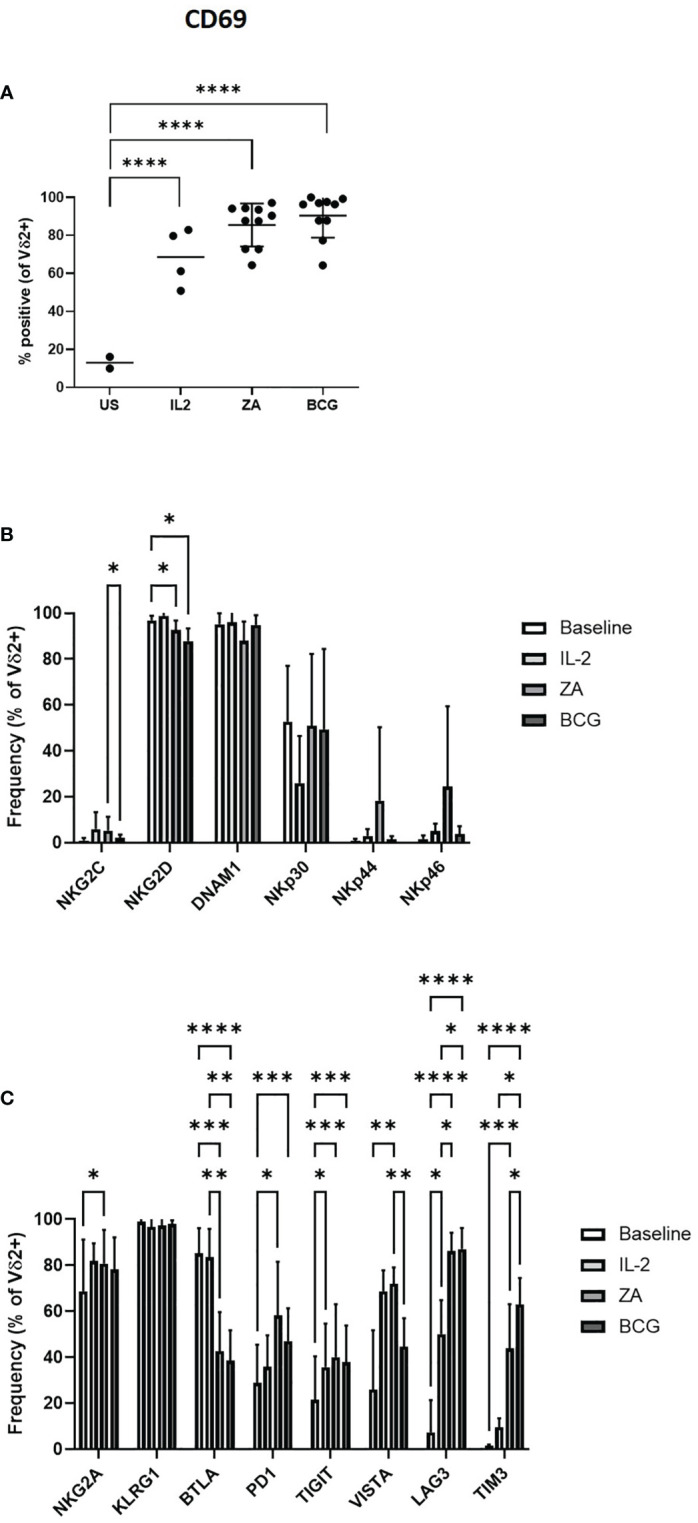
Activation of Vδ2 cells was assessed by flow cytometry of CD69 expression following 24 hours stimulation with IL-2, ZA or BCG both with IL-2 **(A)**. Expression of NK associated activatory markers **(B)** and inhibitory checkpoint receptors **(C)** was determined on Vγ9Vδ2 T-cells in PBMC stimulated for 24 hours with IL-2 alone, ZA or BCG, both with IL-2, using flow cytometry. N=10. *p<0.05, **p<0.005, ***p<0.0005, ****p<0.0001, non-parametric mixed effects analysis with Tukey’s *post hoc* for multiple pairwise comparisons.

The activation of Vδ2+ T-cells by IL-2, ZA and BCG was confirmed by the upregulation of activation marker CD69 (**Figure 2A**). There was no change in expression of NK-associated activatory receptors from baseline with 24-hour stimulation, with the exception of NKG2D which is significantly reduced with both ZA and BCG (**Figure 2B**). There was no difference in expression of activatory markers with stimulation method with the exception of NKG2C which was significantly reduced in BCG activated Vδ2+ T-cells compared to ZA activated Vδ2+ T-cells (**Figure 2B**; **Supplementary Figure 2C**).

There was no change in the expression of inhibitory immune receptor KLRG1 on Vδ2+ T-cells following ZA stimulation (**Figure 2C**). In contrast, there was significantly increased expression of NKG2A PD1, TIGIT, VISTA, LAG3 and TIM3 with ZA stimulation. Similarly, TIGIT, LAG3 and TIM3 are significantly increased with BCG stimulation (**Figure 2C**; **Supplementary Figure 2D**). However, LAG3 and TIM3 were the only receptors significantly increased compared to IL-2 only control. BTLA is the only inhibitory receptor whose expression decreased upon ZA and BCG stimulation, compared to both baseline and IL-2 stimulation (**Figure 2C**). There was reduced expression of VISTA and increased expression of TIM3 on BCG activated Vδ2+ T-cells compared to ZA activated Vδ2+ T-cells (**Figure 2C**).

Furthermore, modulation of activatory and inhibitory receptors following stimulation with ZA and BCG does appear to be specific to Vδ2+ T-cells with minimal changes seen in expression of receptors on CD3+ T-cells and CD56+ NK cells (**Supplementary Figure 3**). Changes in expression of NKG2D, NKp30, LAG3 and TIM3 do reach statistical significance in CD3+ T-cells (**Supplementary Figures 3A, B**). For CD56+ cells DNAM1, NKp44, NKG2A, TIGIT, LAG3 and TIM3 also show statistical significance from baseline (**Supplementary Figures 3C, D**).

### Expression of inhibitor immune checkpoint receptors increases upon Vδ2+ T-cell expansion

After documenting changes in the expression of various activatory and inhibitory receptors following 24-hour Vδ2+ T-cell activation the next aim was to assess whether these changes in expression were maintained over longer periods of stimulation, as seen with Vδ2+ T-cell expansion protocols. Therefore, the expression of activatory and inhibitory receptors was next explored following the expansion of Vδ2+ T-cells in PBMC. Isolated PBMCs were stimulated with IL-2 with and without previously optimised concentrations of ZA, BCG or HK-BCG for 14 days. Expression of activatory and inhibitory markers was assessed by flow cytometry.

**Figure 3:**
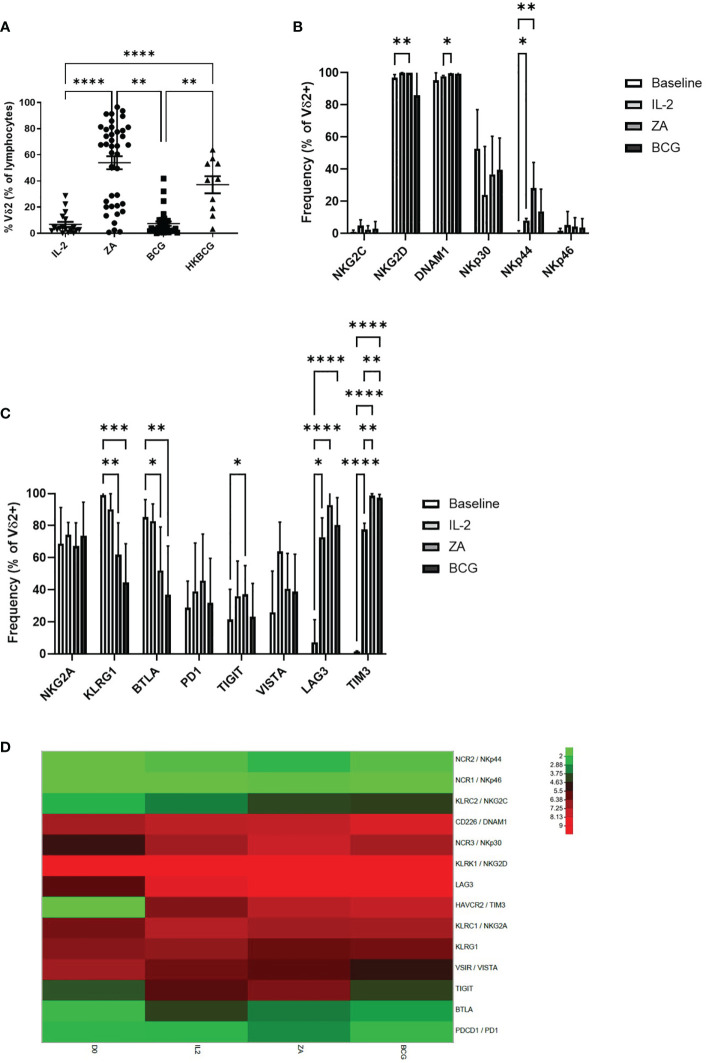
Frequency of Vδ2 cells as a percentage of live cells was assessed by flow cytometry following 14 days stimulation with IL-2 alone or IL-2 with ZA, BCG or HK-BCG. N=10-39, **p<0.005, ****p<0.0001, non-parametric analysis of variance with Tukey’s *post hoc* for multiple pairwise comparisons **(A)**. Expression of NK associated activatory markers **(B)** and inhibitory checkpoint receptors **(C)** was determined on Vγ9Vδ2 T-cells in PBMC stimulated for 14 days with IL-2 alone, ZA or BCG, both with IL-2, using flow cytometry. N=10, *p<0.05, **p<0.005, ***p<0.0005, ****p<0.0001, non-parametric mixed effects analysis with Tukey’s *post hoc* for multiple pairwise comparisons. Expression of activatory and inhibitory receptors was determined in Vγ9Vδ2 T-cells from 6 donors at baseline (D0) and following stimulation for 14 days with IL-2 alone (IL2), ZA (ZA) or BCG (BCG), both with IL-2, using RNAseq **(D)**.

The expansion of Vδ2+ T-cells was also assessed following 14 days stimulation with ZA, BCG or HK-BCG. There were successful expansions of Vδ2+ T-cells when stimulated with ZA and heat-killed BCG (HKBCG), compared to the control IL-2 alone (**Figure 3A**).

High expression of NKG2D and DNAM1 was maintained from baseline and 24 hours activation (**Figure 3B**). NKp44 was increased following 14 days stimulation with ZA (**Figure 3B**). Furthermore, there was no difference in expression of activatory markers following 14 days stimulation with ZA or BCG (**Figure 3C**; **Supplementary Figure 2E**).

There was no change in inhibitory receptors NKG2A, PD1 and VISTA from baseline. However, there was significantly reduced expression of both KLRG1 and BTLA from baseline with both ZA and BCG expansion (**Figure 3C**). TIGIT, LAG3 and TIM3 were significantly increased following 14-day expansion with ZA whereas only LAG3 and TIM3 were significantly increased following 14-day expansion with BCG (**Figure 3C**; **Supplementary Figure 2F**). There was no significant difference in expression of receptors between ZA and BCG-expanded Vδ2+ T-cells suggesting a similar mechanism of activation.

In addition to marker expression as assessed by flow cytometry, corresponding RNAseq analysis of 14 day stimulated Vδ2+ T-cells shows that high expression of NKG2D and DNAM1 is maintained from baseline (**Figure 3D**). Furthermore, the increase in LAG3 and TIM3 was confirmed from baseline with 14-day stimulation (**Figure 3D**). In addition, no clear differences were seen in RNA expression of activatory or inhibitory receptors between ZA and BCG expanded Vδ2+ T-cells (**Figure 3D**).

Following 14-day stimulation there are also differences in expression of activatory and inhibitory receptors on CD3+ T-cells and CD56+ NK cells (**Supplementary Figure 4**). Expression of BTLA is significantly reduced on CD3+ T-cells following ZA and BCG expansion. Furthermore, TIM3 and LAG3 are significantly increased on CD3+ T-cells following ZA and BCG expansion (**Supplementary Figures 4A, B**). Similarly, in CD56+ cells LAG3, TIM3 and NKG2A are significantly increased with both ZA and BCG expansion. While KLRG1 is significantly decreased in CD56+ cells with ZA and BCG expansion. Furthermore, DNAM1, NKp30, NKp44 and NKp46 are significantly increased on CD56+ cells with ZA and BCG expansion (**Supplementary Figures 4C, D**). These changes, largely absent following 24-hour stimulation, suggest an indirect effect of stimulation on other cell populations. High levels of expression of numerous inhibitory receptors may provide a mechanism of immune regulation of Vδ2+ T-cells, important in our understanding when using these cells in immunotherapy.

### Expression of checkpoint receptors and cytokine production is modulated upon culture with tumour cells

Many studies have documented the ability of Vδ2+ T-cells to exert strong anti-tumour responses with both direct cytotoxic function and cytokine production (58, 59). More recently we have shown the ability of BCG to induce a population of Vδ2+ T-cells with superior cytokine and cytolytic mediator production (34). To confirm this, we assessed the ability of Vδ2+ T-cells to lyse various tumour cells and examined the cytokine production of Vδ2+ T-cells in response to stimulation with tumour cells.

**Figure 4:**
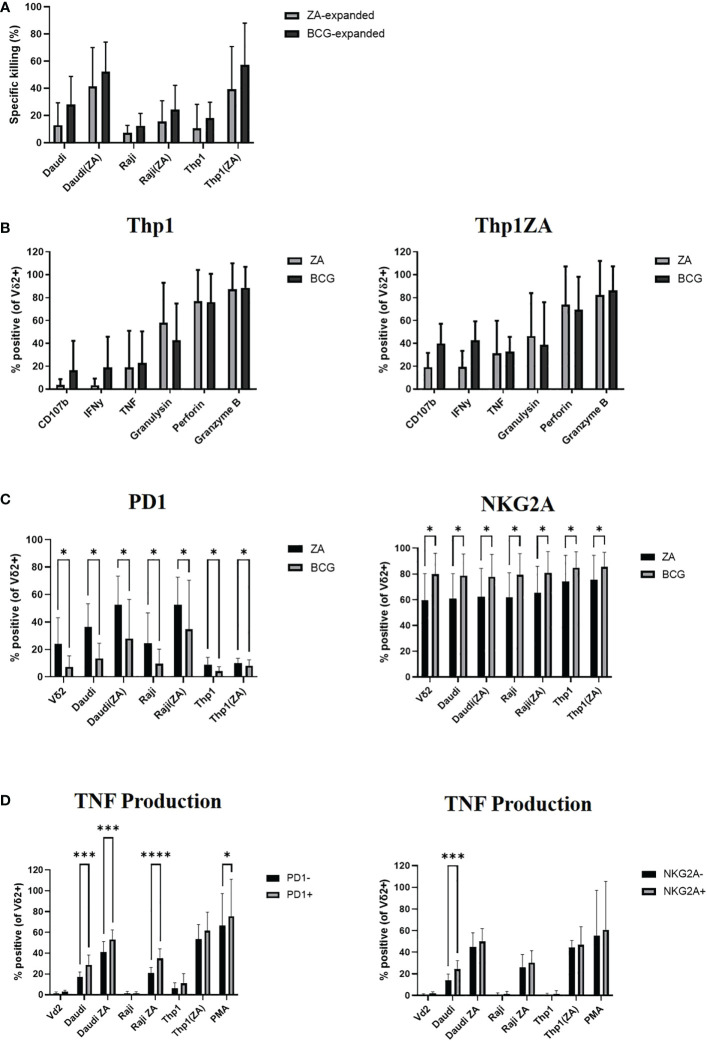
Vδ2 cells, expanded with ZA or HK-BCG, were isolated and cultured with CTFR labelled Daudi, Raji or Thp1 cells, cultured with or without 50μM ZA prior to culture with Vδ2 cells, at a 1:1 ratio. Specific killing of tumour cells was calculated after 16 hours co-culture **(A)**. After 4 hours co-culture the production of CD107b, IFNγ, TNF, granulysin, perforin and granzyme B was assessed by flow cytometry **(B)**, expression of PD1 and NKG2A was assessed **(C)** and production of TNF was assessed on receptor positive and receptor negative cells **(D)**. N=6. *p<0.05, ***p<0.0005, ****p<0.0001, non-parametric analysis of variance with Tukey’s *post hoc* for multiple pairwise comparisons.

Daudi, Raji and Thp1 cells, with and without ZA pre-treatment, were cultured with ZA or HK-BCG expanded Vδ2+ T-cells. As previously shown, there was no significant difference in the killing abilities of Vδ2+ T-cells expanded with ZA or BCG (**Figure 4A**). Next the cytokine production of Vδ2+ T-cells expanded with ZA or HK-BCG was assessed in response to Thp1 cells. There was no significant increase in cytokine production of Vδ2+ T-cells towards Thp1 cells or Thp1 cells pre-treated with ZA (**Figure 4B**).

We next assessed the expression of activatory and inhibitory checkpoint receptors on effector Vδ2+ T-cells following culture with tumour cells. The expression of inhibitory receptors PD1 and NKG2A appears to increase according to tumour sensitivity to killing, likely reflecting the activation of Vδ2+ T-cells in response to tumour cells, (**Figure 4C**). In addition to modulation by activation status/tumour type the expression of inhibitory checkpoint molecules PD1 and NKG2A are also modulated by method of expansion, with significantly decreased expression of PD1 and significantly increased expression of NKG2A on Vδ2+ T-cells expanded with HK-BCG compared to those expanded with ZA (**Figure 4C**). The difference of stimulation on inhibitory receptor expression suggests a possible mechanism of how these two expansion protocols differ in their cytotoxic abilities and may go some way to explaining the differences in cytokine and cytolytic capabilities of BCG and ZA expanded Vδ2+ T-cells. Moreover, Vδ2+ T-cells expressing the high levels of inhibitory receptors PD1 and NKG2A also produced the highest levels of cytokine TNF (**Figure 4D**).

### Effect of immune checkpoint receptor blockade on anti-tumour responses of Vδ2+ T-cells

A mechanism that tumours employ to evade killing is the engagement of checkpoint receptors *via* the expression of checkpoint ligands hence the next aim was to assess the expression of these markers on tumour cells to provide a system for manipulating the effects of these molecules on Vδ2+ T-cells.

**Figure 5:**
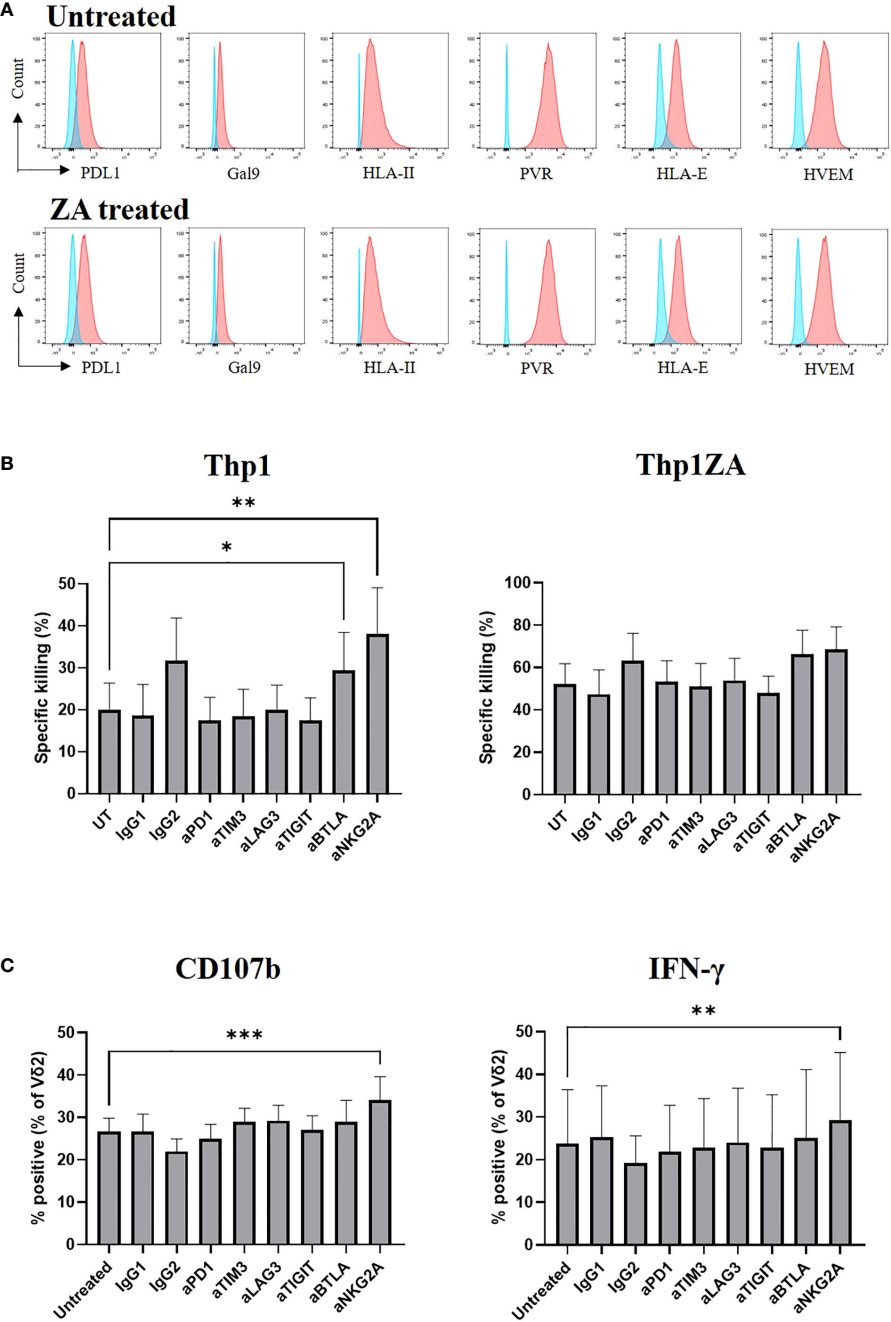
Expression of checkpoint receptor ligands (red) was compared to control (blue) on Thp1 cells with and without 50μM ZA pre-treatment **(A)**. ZA expanded Vδ2 cells were isolated and cultured with CTFR labelled Thp1 target cells, with or without 50μM ZA pre-treatment, at a 1:1 ratio in the presence or absence of 5μg anti-PD1, anti-TIM3, anti-LAG3,anti-TIGIT, anti-BTLA and anti-NKG2A. Specific killing of tumour cells was calculated following 16 hours **(B)** and production of CD107b and IFNγ by Vδ2 cells was assessed by ICS and flow cytometry following 4 hours **(C)**. N=7. *<0.05, mixed effects analysis with Dunnett’s *post hoc* for multiple pairwise comparisons. **p<0.005, ***p<0.0005.

We assessed the expression of ligands towards numerous inhibitory checkpoint receptors by flow cytometry on Thp1 cells at baseline and following pre-treatment with ZA. Thp1 cells express moderate amounts of PDL1, Gal9 and HLA class II and substantial amounts of PVR, HLA-E and HVEM (**Figure 5A**). The expression of checkpoint ligands was not altered following ZA pre-treatment. Therefore, Thp1 cells were used as a model system to interrogate the role of inhibitory checkpoint receptors on Vδ2+ T-cells.

We next carried out functional studies to investigate whether the effector phenotype of Vδ2+ T-cells could be modulated by culture with immune checkpoint inhibitors. ZA expanded Vδ2+ T-cells were cultured with Thp1 cells, with and without ZA pre-treatment, in the presence of blocking antibodies. The blockade of inhibitory receptors PD1, LAG3, TIM3, and TIGIT had no effect on the cytotoxic abilities of Vδ2+ T-cells against Thp1 cells or Thp1 cells with ZA pre-treatment (**Figure 5B**). Blockade of BTLA and NKG2A enhanced the cytotoxic effects of Vδ2+ T-cells against Thp1 cells but any effects were lost upon pre-treatment of Thp1 cells with ZA (**Figure 5B**). Furthermore, there was no influence of blockade of PD1, LAG3, TIM3, TIGIT or BTLA on the production of CD107b and IFN-γ by Vδ2+ T-cells cultured with ZA pre-treated Thp1 cells (**Figure 5C**). Blockade of NKG2A did however significantly enhance production of CD107b or IFN-γ by Vδ2+ T-cells cultured with both Thp1 cells and Thp1 cells pretreated with ZA (**Figure 5C**). Despite high expression of inhibitory receptors on expanded Vδ2+ T-cells the blockade of these molecules shows no clear effect on Vδ2+ T-cell function.

## Discussion

In this current investigation we have explored the role of activatory and inhibitory receptors on Vδ2+ T-cell function and their potential modulation with different routes of Vδ2+ T-cell activation. We also build on our previous findings which highlights BCG as an important mechanism of Vδ2+ T-cell activation which may provide a more physiologically relevant method of expansion which may bypass the potential exhaustion documented with ZA expansion (34, 60).

Vδ2+ T-cells represent a key cell type in immunosurveillance and therefore a crucial potential target of immunotherapy. A key study has found the γδ T-cell to be the cell type that correlates most closely with favourable clinical outcome in cancer patients (15). Despite this, variable responses have been achieved with clinical trials utilising Vδ2+ T-cells, either *via in vivo* phosphoantigen stimulation or *in vitro* expansion followed by adoptive transfer. Many techniques have been tested for Vδ2 expansion such as pulsing with ZA, use of pro-drugs, use of APC along with various cytokines including IL-15 and IL-2 (61–63). Despite this it is likely there is still a large amount of heterogeneity within the Vδ2 population and a potential for high levels of exhaustion (36).

We explore the use of BCG as a method of Vδ2 expansion as this has been shown to result in regression of melanoma lesions and infiltration of Vδ2 cells when injected into lesions (13). Despite the previously reported differences in cytolytic profile of Vδ2 cells in response to BCG we describe no significant difference in activatory marker or inhibitory marker expression following BCG activation and expansion compared to ZA. We do describe a difference in expression of PD1 and NKG2A following culture with tumour cells which suggests these stimuli have different mechanisms of action. Indeed, studies have shown differential roles of accessory cells between ZA, HMBPP and BTN3A1 in Vδ2 activation (64). Therefore, the differences in activation and inhibition warrant further understanding.

Unlike other CD3+ T-cells Vδ2+ T-cell activation can be achieved *via* stimulation through the TCR and BTN3A1/BTN2A1 or *via* stress ligands recognised by NCR receptors. However, concurrent stimulation through inhibitory checkpoint receptors may block activation and subsequent cytotoxicity. Therefore, it is likely a balance between these activatory and inhibitory receptors may control the outcome upon tumour encounter. First, we have shown that Vδ2+ T-cells in circulation express various activation markers. Vδ2+ T-cell recognition has been widely documented to be due to recognition of phosphoantigens *via* BTN3A1 and the TCR (6–8). Recognition also occurs *via* NKG2D and the recognition of stress ligands MICA/B and ULBPs (11, 65, 66). The relative contributions of these receptors is debated, with some showing TCR independent recognition with significant reduction in Vδ2+ T-cell mediated killing with NKG2D blockade (11, 65, 67). Others suggest that NKG2D acts as a costimulatory receptor *via* modulating early TCR signals (68, 69). Others highlight roles for both NKG2D and TCR with the perforin granzyme pathway as the main mechanism of cytotoxicity (70).

Further evidence for the costimulatory receptor theory comes from a study showing DNAM1 is constitutively expressed on circulating Vδ2+ T-cells and maintained upon activation, findings which have been recapitulated in our study. Tumour lysis could be inhibited by anti-DNAM1 with NKG2D blockade providing complementary contribution to cytotoxicity (71). The high levels of both NKG2D and DNAM1 on both circulating and activated Vδ2+ T-cells found in this study suggest crucial roles for these receptors in recognition. This can be confirmed as the investigation into the use of Vδ2 cells modified with the addition of NKG2D RNA CAR revealed enhanced cytoltic activity, an effect that was enhanced with the addition of ZA (46).

Expression of NCRs may also play a costimulatory role in Vδ2+ T-cell recognition. In this study we see limited expression of NKp44 and NKp46 on Vδ2+ T-cells but intermediate expression of NKp30. There was no clear upregulation of these markers following stimulation in our study despite expression of these molecules being documented to be upregulated on Vδ1+ T-cells with stimulation with IL-2, IL-15 or TCR (72, 73). NKp30 and NKp46 are enhanced on NK cells exposed my mycobacterium tuberculosis infected monocytes however NKp44 is enhanced on NK cells following BCG stimulation and not by stimulation with mycobacterium tuberculosis infected monocytes (74, 75). This suggests these receptors may play a differential role in recognition of mycobacteria. In this study however, we saw no upregulation of NKp44 on Vδ2+ T-cells expanded with BCG. No differences in NKp44 or NKp46 expression was seen between ZA-expanded and BCG-expanded Vδ2+ T-cells, as previously documented (76).

It is likely that the mechanism of Vδ2+ T-cell recognition involves a complex interplay between a combination of activatory receptors, depending on the ligands present. In this study we did not explore the role of activatory receptors typically found on αβ T-cells such as TNFR-family receptors CD27 and 4-1BB. These molecules have been documented as modulators of Vδ2 activation with roles in proliferation, survival and secretion of inflammatory cytokines and as such these require further investigation into their contribution to Vδ2 activation (77–81). In addition to the expression of stress ligands on tumour cells and recognition by activatory markers, Vδ2 cells may also recognize tumour cells by the downregulation of MHC class I and subsequent activation of KIR and LILR. LILR have been documented to be expressed on Vδ2 cells associated with presence of infection both with CMV and mycobacteria (82, 83). KIR have also been documented to be expressed on Vδ2 cells, particularly more cytolytic CD16+ cells. This expression of KIR likely explains the observation in **Figure 4** of increased killing of Daudi cells lacking MHC class I compared to Raji and Thp1 cells which require other mechanisms of recognition (84–87). These additional receptors are important to consider as it is likely that this highly diverse combination of receptors plays a role in the suboptimal use of Vδ2+ T-cells in immunotherapy.

Next, we investigated the expression of inhibitory immune checkpoint receptors on Vδ2+ T-cells. Inhibitory immune checkpoint receptors are well documented on CD4 and CD8 T-cells with roles in suppression of proliferation, activation and cytokine production (88). Less is known about the expression of inhibitory checkpoint receptors on Vδ2+ T-cells and their role in regulation of these cells. Inhibitory receptors PD1, TIM3, LAG3 and BTLA have been documented to be expressed on Vδ2+ T-cells with upregulation upon stimulation (49, 55, 89–92). In contrast we document no change in PD1 expression with mycobacterial or phosphoantigen stimulation. One study documents minimal expression of PD1 on Vδ2+ T-cells which increases following 3 days stimulation with HMBPP followed by a gradual decline (49). This suggests that the time points investigated in this study may have missed any increase in expression of PD1. We document marked decrease in BTLA expression, something which has been documented in the literature, and maximal expression of both TIM3 and LAG3 following stimulation and these molecules, along with PD1, on γδ T-cells have been shown to associate with earlier relapse and shorter overall survival in melanoma patients suggesting these molecules may play a role in Vδ2+ T-cell regulation (17, 93).

NKG2A is a recently emerging checkpoint molecule shown to be expressed on CD8+ T-cells and NK cells with blockade potentiating effector functions (94, 95). We see high levels of expression on Vδ2+ T-cells providing a new cell type which would be targeted by such interventions. In the aforementioned studies blockade of NKG2A increased the frequencies of CD107 and IFN-γ by NK and CD8 T-cells so it will be of interest to study the impact of NKG2A blockade on Vδ2 activation and function.

Finally, we examined the impact of blockade of immune checkpoint receptors on Vδ2+ T-cell function. We saw no difference in Vδ2+ T-cell cytotoxicity nor any difference in cytokine production against Thp1 cells which express intermediate levels of all immune checkpoint ligands. Other studies have demonstrated that blockade of anti-PD1, anti-BTLA and anti-TIM3 result in enhanced proliferation and prevention of apoptosis (53, 89, 93). Any effects of the blockade of immune checkpoints on Vδ2+ T-cells may be limited to proliferation or cell death. Another plausible reason for this difference is the expression of immune checkpoints and corresponding ligands on tumour cells as expression of certain ligands in our model were limited, as in the case of PD1. Despite studies documenting a role of checkpoint inhibition in Vδ2+ T-cells there is still debate about the significance of this approach in these cells. Some have found no effect of blocking PD1 in cell lines expressing high PDL1 (54). One possibility may be that strong TCR signalling or the additional effect of NKG2D co-signalling may overcome any inhibitory effect of PD1. We also document only low levels of PD1 expression suggesting there is not enough expression for this to be an effective checkpoint. Others have found that a combination of checkpoint blockade has better effect in Vδ2+ T-cells. Blockade of PD1 alone had no effect on cytokine production of Vδ2+ T-cell however when combined with anti-TIM3 elevated cytokine production was observed suggesting PD1 alone is insufficient to correct functional impairment (55).

Due to the high levels of TIM3, LAG3 and NKG2A found upon Vδ2+ T-cells following expansion it is crucial to explore combinations of these checkpoint receptors in Vδ2+ T-cell function. As treatment with ipilimumab has been shown to result in higher proportions of Vδ2 cells and those patients with poor response had lower frequencies of Vδ2 cells the combination of Vδ2 immunotherapy with checkpoint blockade would be envisaged to be beneficial in anti-tumour therapy. As such, trials are ongoing into the combination of Vδ2 activation with anti-BTN3A1 in combination immune checkpoint blockade (NCT04243499) (39). Like ZA, this molecule has been shown to enhance the sensitivity of tumour cells to Vδ2 killing and enhances Vδ2 production of IFN-γ, TNF, granzyme B and perforin.

Overall, we have found a high level of expression of activatory molecules and inhibitory immune checkpoints on Vδ2+ T-cells. Levels of these markers are modulated upon phosphoantigen and mycobacterial activation and provide a crucial target of tumour cells to regulate Vδ2+ T-cell responses. This work suggests crucial combinations of immune checkpoint blockade that would be useful to improve the success of Vδ2+ T-cell in immunotherapy.

## Data availability statement

The data discussed in this publication have been deposited in NCBI's Gene Expression Omnibus and are accessible through GEO Series accession number GSE221563 (https://www.ncbi.nlm.nih.gov/geo/query/acc.cgi?&acc=GSE221563).

## Ethics statement

The studies involving human participants were reviewed and approved by St George’s Research Ethics Committee. The patients/participants provided their written informed consent to participate in this study.

## Author contributions

LR conducted the laboratory work, analyses and drafted the manuscript. JC contributed to laboratory work and analysis. MB-S contributed to study conception and design. All authors contributed to the article and approved the submitted version.

## Supplementary material

Supplementary Figure 1 Gating strategy for activatory and inhibitory receptor expression. Doublets were excluded by SSC-A versus SSC-W and lymphocytes gated based on SSC-A versus FSC-A. Live cells were gated as negative for viability dye Zombie Aqua. From the live population CD3 was gated against activatory and inhibitory markers including NKG2A, KLRG1, BTLA, PD1, TIGIT, VISTA, LAG3, TIM3, NKG2C, NKG2D, DNAM1, NKp30, NKp44 and NKp46. Positivity was determined by quadrant gates set based on the expression within live cells using a contour plot with level 5%. To identify cell subsets CD3 positive and CD3 negative populations were gated. From the CD3 positive cells T-cells and Vδ2 cells were gated based on Vδ2 expression. From the CD3 negative population NK cells were gated based on positive expression of CD56. The positive quadrant gates for activatory and inhibitory markers were then copied onto the relevant cell sub-populations.

Supplementary Figure 2 Expression of NK associated activatory receptors and inhibitory checkpoint receptors was determined in Vγ9Vδ2+ cells in PBMCs from healthy donors at base line **(A and B)**, following 24 hours stimulation with IL-2 alone, ZA or BCG, both with IL-2 **(C and D)** or following 14 days expansion with IL-2 alone, ZA or BCG, both with IL-2 **(E and F)**. N=10. *p<0.05, **p<0.005, ***p<0.0005, ****p<0.0001, non-parametric analysis of variance with Tukey’s *post hoc* for multiple pairwise comparisons.

Supplementary Figure 3 Expression of NK associated activatory markers on CD3+ T-cells **(A)** CD56+ NK cells **(B)** and inhibitory checkpoint receptors on CD3+ T-cells **(C)** and CD56+ NK cells **(D)** was determined in PBMC stimulated for 24 hours with IL-2 alone, ZA or BCG, both with IL-2, using flow cytometry. N=10, *p<0.05, **p<0.005, ***p<0.0005, ****p<0.0001, non-parametric mixed effects analysis with Tukey’s *post hoc* for multiple pairwise comparisons.

Supplementary Figure 4 Expression of NK associated activatory markers on CD3+ T-cells **(A)** CD56+ NK cells **(B)** and inhibitory checkpoint receptors on CD3+ T-cells **(C)** and CD56+ NK cells **(D)** was determined in PBMC stimulated for 14 days with IL-2 alone, ZA or BCG, both with IL-2, using flow cytometry. N=10, *p<0.05, **p<0.005, ***p<0.0005, ****p<0.0001, non-parametric mixed effects analysis with Tukey’s *post hoc* for multiple pairwise comparisons.
