# Supplementary figures and images for: Releasing the restraints of Vγ9Vδ2 T-cells in cancer immunotherapy
Source: Front Immunol. 2023 Jan 13;13:1065495. doi: 10.3389/fimmu.2022.1065495 (PMC9880221; doi:10.3389/fimmu.2022.1065495)

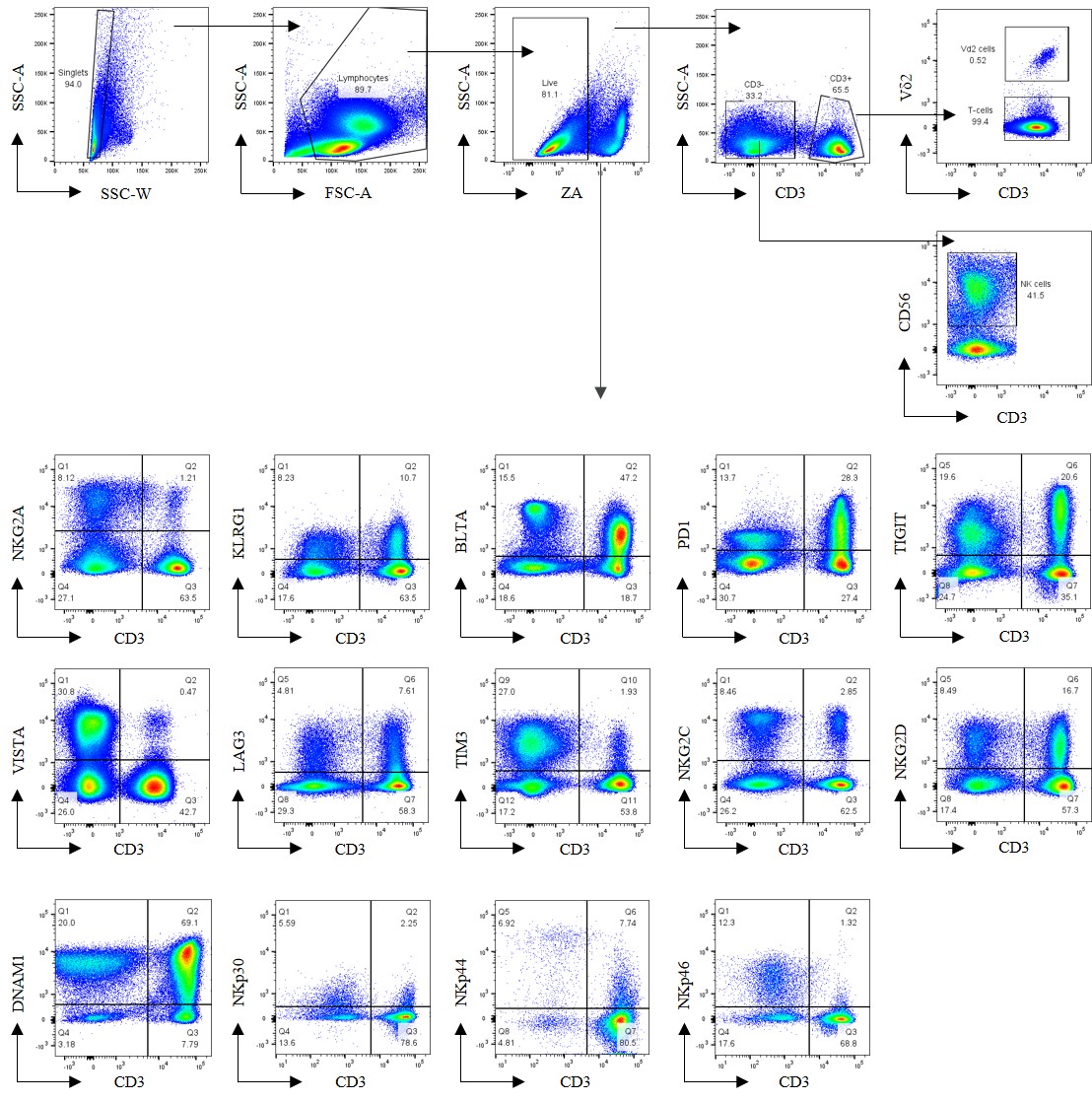

Supplement: Supplementary Figure 1 — Gating strategy for activatory and inhibitory receptor expression. Doublets were excluded by SSC-A versus SSC-W and lymphocytes gated based on SSC-A versus FSC-A. Live cells were gated as negative for viability dye Zombie Aqua. From the live population CD3 was gated against activatory and inhibitory markers including NKG2A, KLRG1, BTLA, PD1, TIGIT, VISTA, LAG3, TIM3, NKG2C, NKG2D, DNAM1, NKp30, NKp44 and NKp46. Positivity was determined by quadrant gates set based on the expression within live cells using a contour plot with level 5%. To identify cell subsets CD3 positive and CD3 negative populations were gated. From the CD3 positive cells T-cells and Vδ2 cells were gated based on Vδ2 expression. From the CD3 negative population NK cells were gated based on positive expression of CD56. The positive quadrant gates for activatory and inhibitory markers were then copied onto the relevant cell sub-populations. [file Image_1.jpeg]

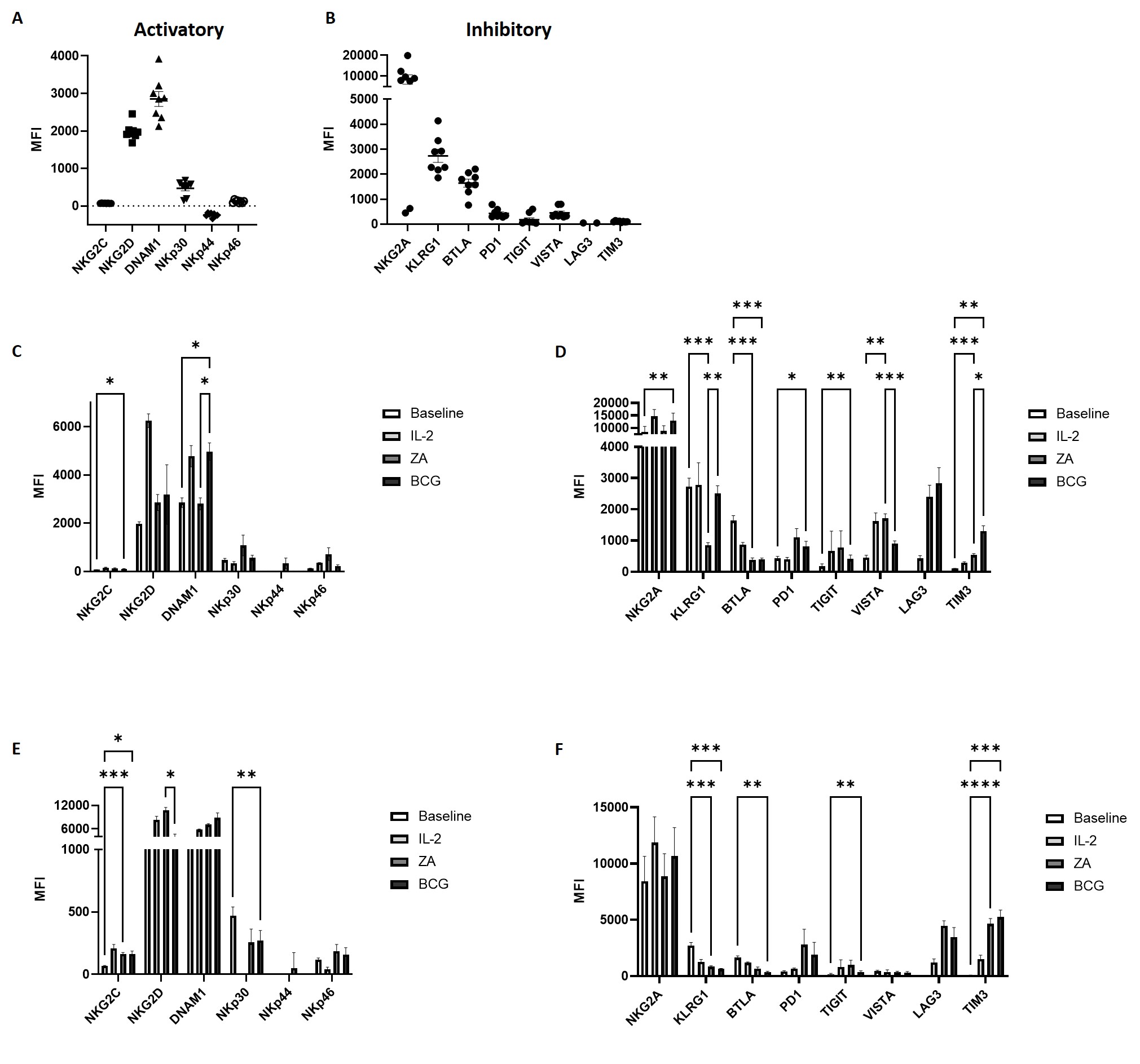

Supplement: Supplementary Figure 2 — Expression of NK associated activatory receptors and inhibitory checkpoint receptors was determined in Vγ9Vδ2+ cells in PBMCs from healthy donors at base line (A and B), following 24 hours stimulation with IL-2 alone, ZA or BCG, both with IL-2 (C and D) or following 14 days expansion with IL-2 alone, ZA or BCG, both with IL-2 (E and F). N=10. *p<0.05, **p<0.005, ***p<0.0005, ****p<0.0001, non-parametric analysis of variance with Tukey’s post hoc for multiple pairwise comparisons. [file Image_2.jpeg]

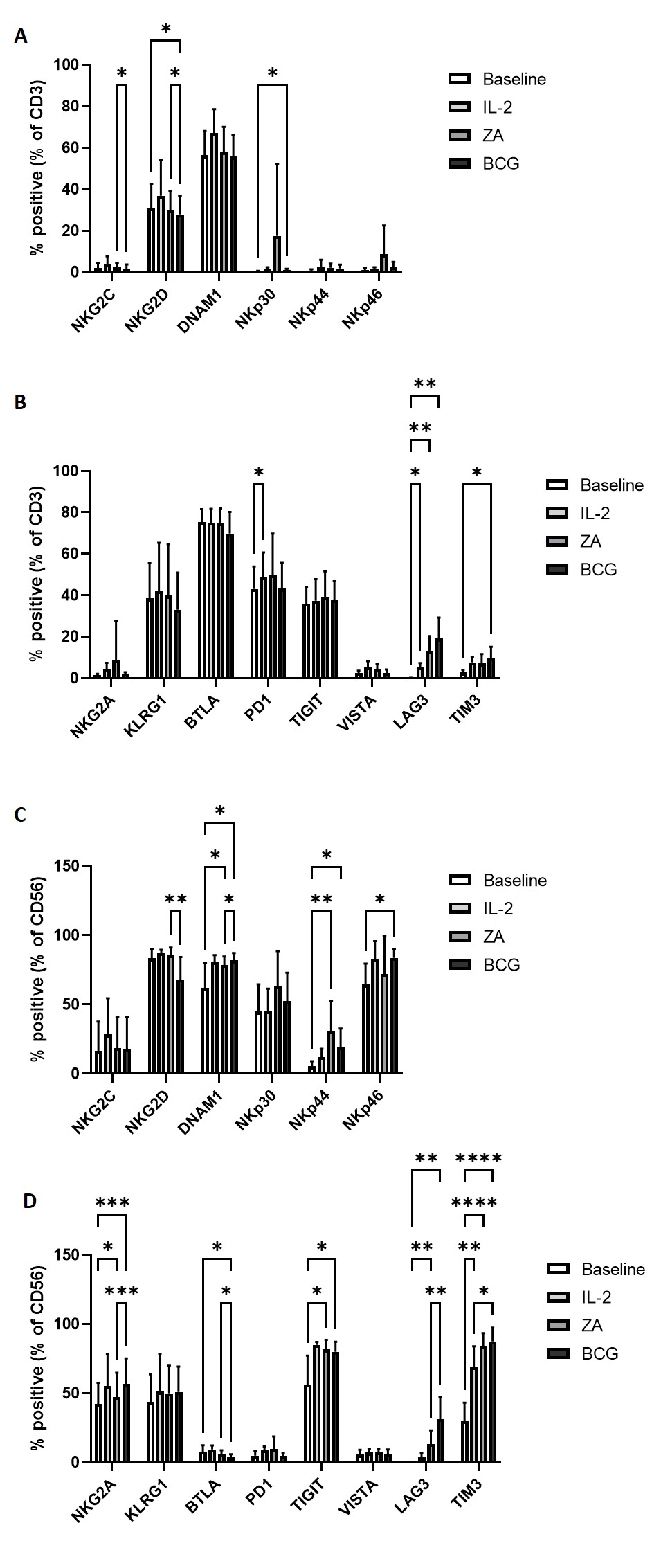

Supplement: Supplementary Figure 3 — Expression of NK associated activatory markers on CD3+ T-cells (A) CD56+ NK cells (B) and inhibitory checkpoint receptors on CD3+ T-cells (C) and CD56+ NK cells (D) was determined in PBMC stimulated for 24 hours with IL-2 alone, ZA or BCG, both with IL-2, using flow cytometry. N=10, *p<0.05, **p<0.005, ***p<0.0005, ****p<0.0001, non-parametric mixed effects analysis with Tukey’s post hoc for multiple pairwise comparisons. [file Image_3.jpeg]

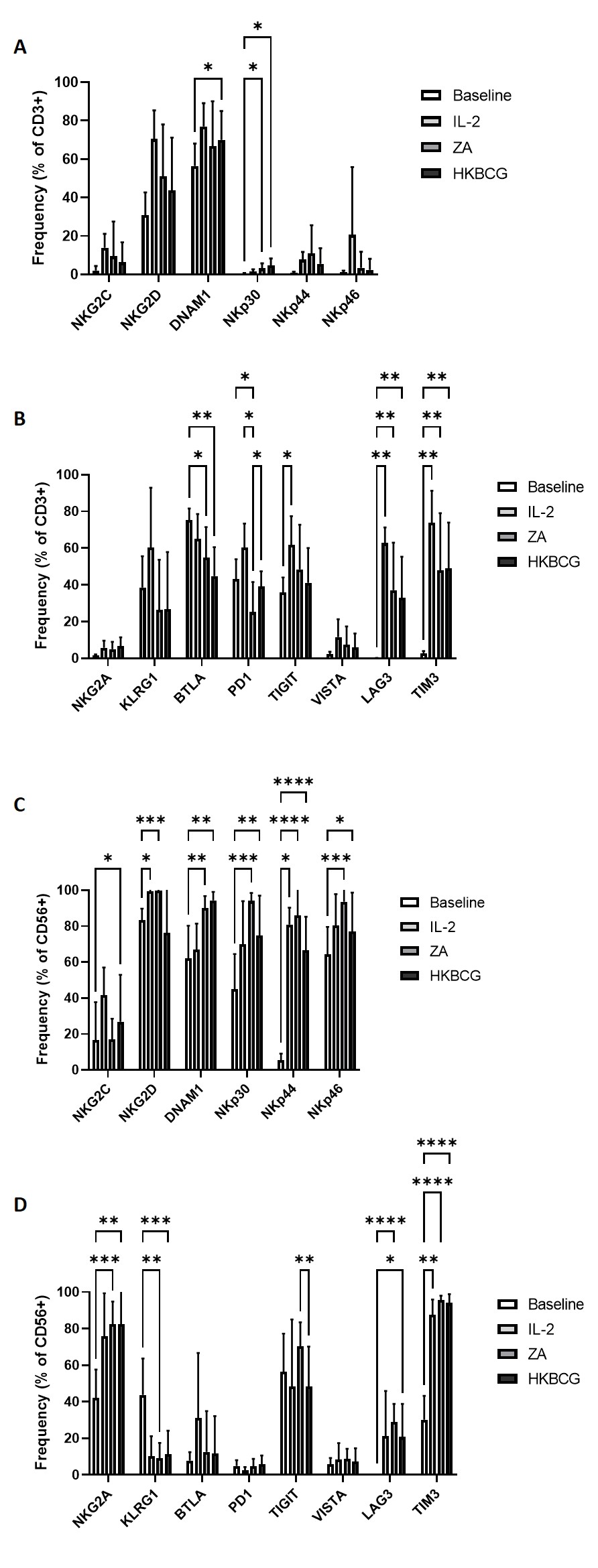

Supplement: Supplementary Figure 4 — Expression of NK associated activatory markers on CD3+ T-cells (A) CD56+ NK cells (B) and inhibitory checkpoint receptors on CD3+ T-cells (C) and CD56+ NK cells (D) was determined in PBMC stimulated for 14 days with IL-2 alone, ZA or BCG, both with IL-2, using flow cytometry. N=10, *p<0.05, **p<0.005, ***p<0.0005, ****p<0.0001, non-parametric mixed effects analysis with Tukey’s post hoc for multiple pairwise comparisons. [file Image_4.jpeg]
